# *In planta *expression of *A. cellulolyticus *Cel5A endocellulase reduces cell wall recalcitrance in tobacco and maize

**DOI:** 10.1186/1754-6834-4-1

**Published:** 2011-01-26

**Authors:** Roman Brunecky, Michael J Selig, Todd B Vinzant, Michael E Himmel, David Lee, Michael J Blaylock, Stephen R Decker

**Affiliations:** 1 Biosciences Center, National Renewable Energy Laboratory, 1617 Cole Boulevard, MS 3323, Golden, CO 80401, USA; 2 Edenspace Systems Corporation, 3810 Concorde Parkway, Suite 100, Chantilly, VA 20151-1131, USA

## Abstract

The glycoside hydrolase family 5 endocellulase, E1 (Cel5A), from *Acidothermus cellulolyticus *was transformed into both *Nicotiana tabacum *and *Zea mays *with expression targeted to the cell wall under a constitutive promoter. Here we explore the possibility that *in planta *expression of endocellulases will allow these enzymes to access their substrates during cell wall construction, rendering cellulose more amenable to pretreatment and enzyme digestion. Tobacco and maize plants were healthy and developed normally compared with the wild type (WT). After thermochemical pretreatment and enzyme digestion, transformed plants were clearly more digestible than WT, requiring lower pretreatment severity to achieve comparable conversion levels. Furthermore, the decreased recalcitrance was not due to post-pretreatment residual *E1 *activity and could not be reproduced by the addition of exogenous *E1 *to the biomass prior to pretreatment, indicating that the expression of *E1 *during cell wall construction altered the inherent recalcitrance of the cell wall.

## Background

Plant cell walls are composed of three basic structural biomolecules: cellulose, hemicellulose and lignin. The conversion of cellulose and hemicellulose to sugar is the primary step in the fermentative conversion of biomass to fuels and chemicals [1]. Natural enzymatic digestion of plant matter is a slow and complex process which must become more cost-effective if commercial biofuel production is to become a reality. Cellulases must penetrate hemicellulose and lignin barriers to bind and digest the cellulose. It is the relatively poor accessibility of substrates to enzymes due to the strong associations between plant cell wall components that explains most of this recalcitrance and makes costly thermochemical pretreatments necessary.

Approaches to the deconstruction of plant cell walls to fermentable free sugars typically employ various thermochemical pretreatments followed by an enzymatic hydrolysis step. Whereas several studies have examined the potential to produce and harvest active cellulase from plants, no work has been reported examining the effect of this expression on the amenablilty of these plants to biomass conversion [2-12]. Pretreatment of the biomass through heat, physical force and/or chemical catalysts reduces the inherent strength of the cell wall by altering the physical arrangement of these three components and breaking chemical bonds that give these structures strength. Pretreatment renders the cellulose and hemicellulose components more amenable to conversion to monomeric sugar by enzymes [13]. This transformation of biomass to a more maleable form, however, comes at a cost. The severity required to achieve this reduction in recalcitrance is often high enough to negatively affect the economics of the conversion process through increased capital construction costs, degradation products and inhibitor formation [14,15].

The *A. cellulolyticus E1 *endoglucanase is a well-characterized glycoside hydrolase family 5 (cel5A) endocellulase known to be thermally tolerant and generally very stable over broad ranges of pH [16]. When added exogenously to biomass prior to pretreatment, it does not have any effect on pretreatability or post-pretreatment enzymatic digestibility. We expressed the *Acidothermus cellulolyticus *glycoside hydrolase family 5 endoglucanase (*E1*) in both *Nicotiana tabacum *and *Zea mays *and targeted its expression to the cell wall under a constitutive promoter. Instead of assuming adequate penetration into mature cell walls, we theorized that *in planta *expression would allow the enzyme to access a wider range of cell wall components and compartments as they were being constructed. Because *E1 *has an optimum temperature of approximately 80°C, we suspected that activity during plant growth would be limited and would have minor if any impact on plant phenotype and health.

## Results

### Estimation of *E1*cd in transgenic stover and tobacco

**Figure 1:**
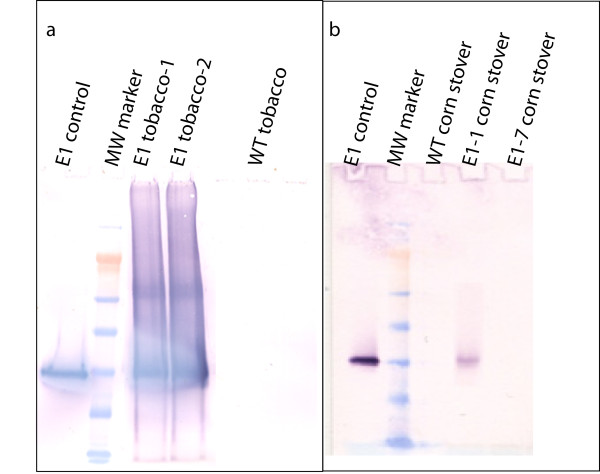
**E1 expression *in planta***. Western blot analysis of wild-type (WT) and E1-transformed tobacco and corn stover.

**Figure 2:**
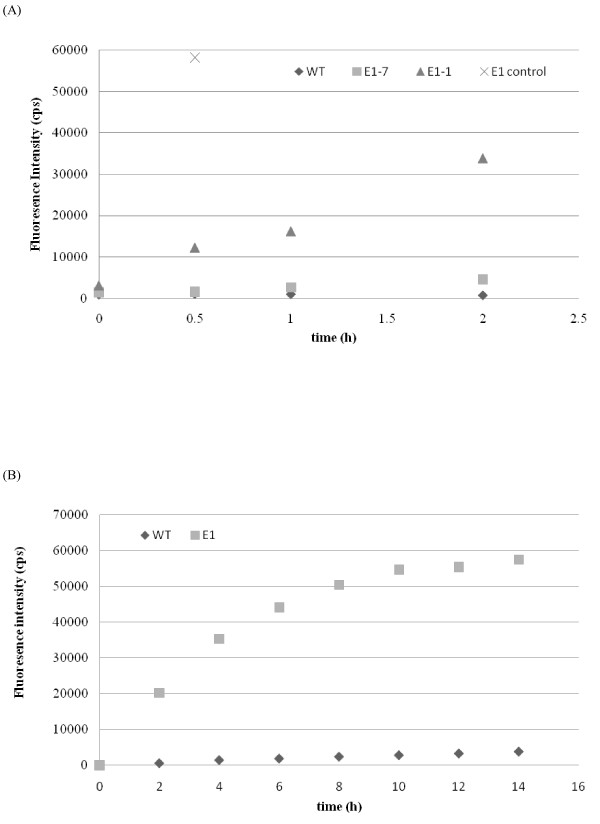
**E1 activity from plant extracts**. **(a) **WT and E1 corn stover and **(b) **tobacco were extracted and assayed for activity on 4-methylumbelliferyl-β-D-cellobiose (MUC) substrate.

Both antibody- and activity-based assays were used to estimate the content of *E1*cd in stover and tobacco (data not shown). Initial Western blot analyses performed by extracting ~4 mg of milled stover with 50 μL of either 100% ethylene glycol at 80°C for 2 hours or 100% ethylene glycol followed by buffer (20 mM sodium acetate, 100 mM NaCl, pH 5.0) showed no bands (data not shown). Increasing the extraction severity by direct boiling of biomass in NuPAGE lithium dodecyl sulfate (LDS) sample buffer (Invitrogen, Carlsbad, CA, USA) gave a strong band for the *E1*-1 stover sample which was the correct molecular weight for *E1*cd (Figure 1). In addition, for stover, a serial twofold dilution series Dot blot estimated the *E1*cd content of *E1*-1 to be approximately 3 ng/mg biomass (data not shown), which was a reasonable approximation for the Western blot analysis as well. The *E1*-7 sample was not detected by either antibody method; however, the 4-methylumbelliferyl-β-D-cellobiose (MUC) activity assay estimated the *E1*-7 level to be about 10-fold less than that of *E1*-1 (Figure 2). The MUC assay revealed *E1*cd levels of approximately 0.3 ng/mg biomass for *E1*-1 and 0.03 ng/mg biomass for *E1*-7. Wild-type stover showed no presence of *E1 *by either method. For tobacco, *E1*cd levels were approximately 1,000-fold higher than those in the transgenic stover. Western blot analysis estimated *E1*cd levels in tobacco to be approximately 3.1 μg/mg biomass. The higher level of *E1 *in tobacco is likely due to the use of the cauliflower mosaic virus (CaMV) promoter to drive expression in both maize and tobacco, as CaMV has been shown to be more active in dicotyledon plants.

### Pretreatment and saccharification of biomass

**Figure 3:**
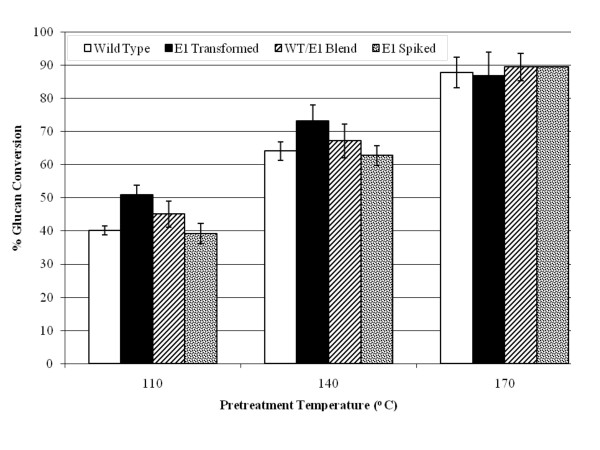
**Saccharification of transgenic tobacco**. Saccharification (72 hours at 50°C) of transgenic tobacco pretreated at 110°C, 140°C and 170°C with 100 mg/g cellulose loading of the commercial cellulase Spezyme CP at 100 mg of Spezyme per gram of biomass. Error bars represent standard deviations of triplicate sample analyses.

*E1*-expressing tobacco plants were pretreated in 1% (wt/vol) sulfuric acid for 10 minutes at three different temperatures, 110°C, 140°C and 170°C, resulting in a range of severities. Figure 3 illustrates that at 110°C and 140°C pretreatment temperatures, *E1*cd-transformed tobacco was about 10% more enzymatically digestible compared to the wild type or to wild-type biomass with exogenous *E1*cd added. Interestingly, the mixed wild-type/*E1 *sample is right between the *E1*cd-containing sample and the wild-type sample, supporting the observation that *in planta*-expressed *E1*cd required less severe pretreatment than the wild-type plant to achieve comparable sugar release. We also observed that at the highest pretreatment severity, 170°C, all of the samples exhibited a similar level of digestion. Presumably, at severe pretreatment levels, the improved conversion of the *E1*-containing tobacco becomes irrelevant as the conversion of the materials approaches a theoretical maximum.

**Figure 4:**
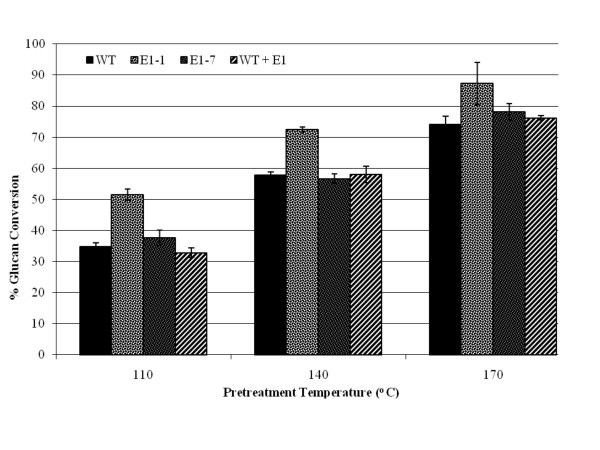
**Saccharification of transgenic corn stover at 15 mg/g loading**. Saccharification (72 hours at 50°C) of transgenic corn stover pretreated at 110°C, 140°C and 170°C with 15 mg/g cellulose loading of the commercial cellulase Spezyme CP at 15 mg of Spezyme per gram of biomass. Error bars represent standard deviations of triplicate sample analyses.

**Figure 5:**
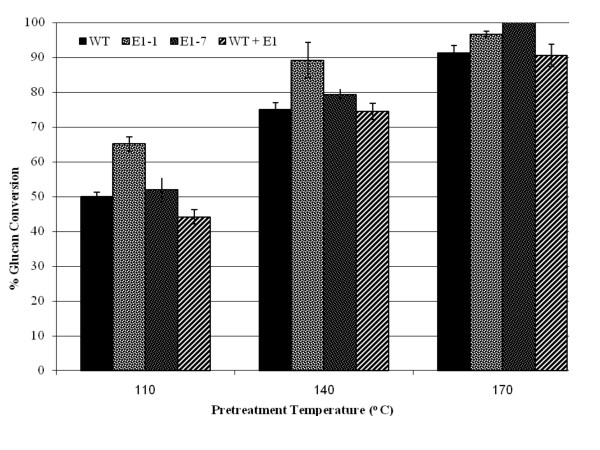
**Saccharification of transgenic corn stover with 100 mg/g loading**. Saccharification (72 hours at 50°C) of transgenic corn stover pretreated at 110°C, 140°C and 170°C with 100 mg/g cellulose loading of the commercial cellulase Spezyme CP at 100 mg of Spezyme per gram of biomass. Error bars represent standard deviations of triplicate sample analyses.

We pretreated wild-type and two E1cd-expressing stover samples (E1-1 and E1-7), as well as wild-type stover treated with exogenously added E1cd, using diluted sulfuric acid at three different pretreatment severities. Figures 4 and 5 illustrate two effects of E1cd expression in maize. First, at the low enzyme loading of 15 mg/g Spezyme CP (Genencor International, Palo Alto, CA, USA) (Figure 4), under all three pretreatment severities, the high-level E1cd-expressing stover (E1-1) appeared to achieve 10% to 15% higher conversions compared to the wild-type and E1cd-treated wild-type samples. Second, the level of increased conversion was correlated to the level of E1cd in the stover, as the E1-7 sample with a very low level of E1cd had a marginally higher conversion rate compared to the wild type. In contrast, the E1-1 sample, which showed a significantly higher E1cd expression level than E1-7, appeared to achieve markedly higher conversions than the wild type. Also, we noted that adding exogenous E1cd to the wild-type stover before pretreatment did not increase its post-pretreatment enzymatic digestibility.

**Figure 6:**
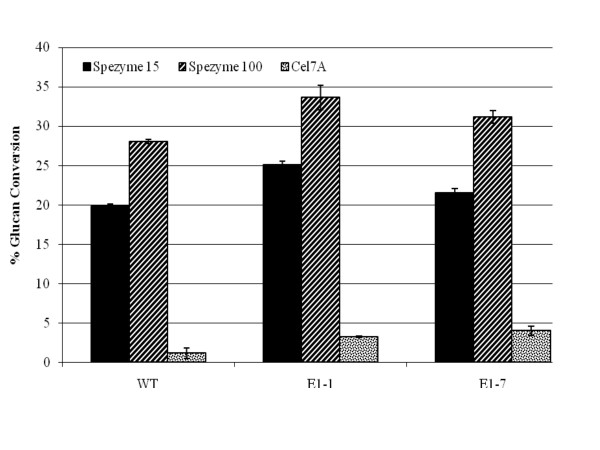
**Saccharification of unpretreated corn stover**. Saccharification (24 hours at 50°C) of unpretreated corn stover with two loadings of Spezyme CP (15 and 100 mg/g cellulose) or *T. reesei *Cel7a (15 mg/g cellulose). Error bars represent standard deviations of triplicate sample analyses.

**Figure 7:**
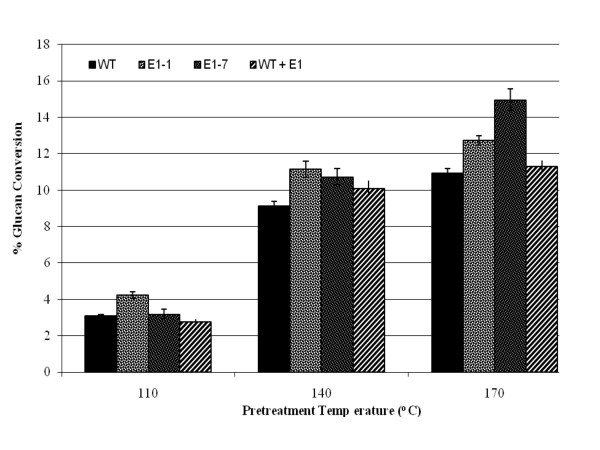
**Glucan conversion of E1-enhanced corn stover compared to WT**. Twenty-four-hour glucan conversion of E1-enhanced corn stover compared to WT using 15 mg/g Cel7A. Error bars represent standard deviations of triplicate sample analyses.

When digestions utilizing commercial cellulase loadings of 15 mg/g to those loaded at 100 mg/g (Figures 4 and 5) were compared, the E1cd-stover sample with the high E1 expression level (E1-1) appeared to show, at 15 mg/g loading, a comparable or higher rate of conversion compared to the wild-type stover treated with the 100 mg/g loading under the same pretreatment conditions. We also assessed the enzymatic conversion of the corn stover without pretreatment or preincubation (Figure 6) and again observed that the E1-1 corn stover appeared to be 5% to 8% more digestible than the wild-type control. Utilizing an exoglucanase (Cel7A) instead of a commercial cellulase yielded comparable conversion levels for both the high and low E1 samples, both of which appeared to have a higher glucan conversion compared to the control and the control plus exogenous E1 (Figure 7).

### Imaging of E1 maize and tobacco

**Figure 8:**
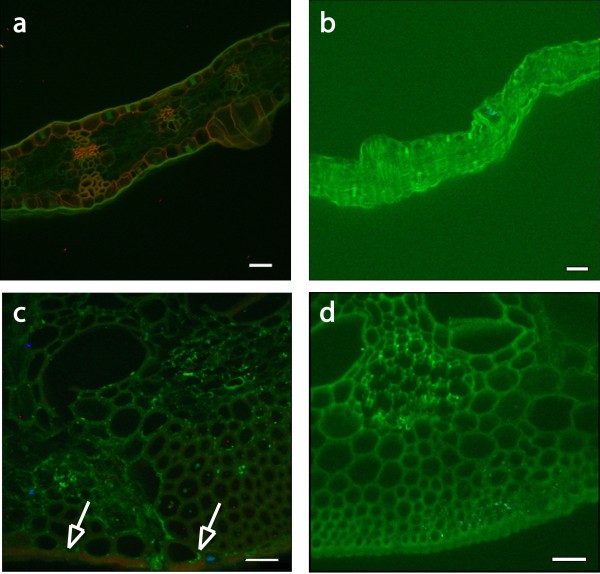
**Localization of E1 in transgenic corn stover. Confocal microscopic images of sectioned WT and E1-containing corn stover**. All images were obtained by using immunofluorescence confocal laser microscopy. An *E1cd *primary antibody and an Alexa Fluor 488 antimouse secondary antibody were used and spectrally deconvoluted to show antibody in red and nonspecific autofluoresence in green. **(a) **Original magnification ×200 image of *E1*-transgenic maize leaf tissue indicating that the transgenic tissue exhibits a broad expression of *E1 *throughout the plant. Scale bar, 50 μm. **(b) **Original magnification ×200 confocal microscopic image of leaf tissue from a WT control corn stover stalk showing no expression of E1 enzyme. Scale bar, 50 μm. **(c) **Original magnification ×600 image of *E1*-transgenic maize stem tissue. This image indicates that *E1*-transgenic tissue exhibits specific expression of *E1 *in the lignified sclerenchyma cells in the bottom portion of the image. Scale bar, 25 μm. **(d) **Original magnification ×600 confocal microscopic image of stem tissue from a WT control corn stover stalk showing no expression of *E1 *enzyme. Scale bar, 25 μm.

**Figure 9:**
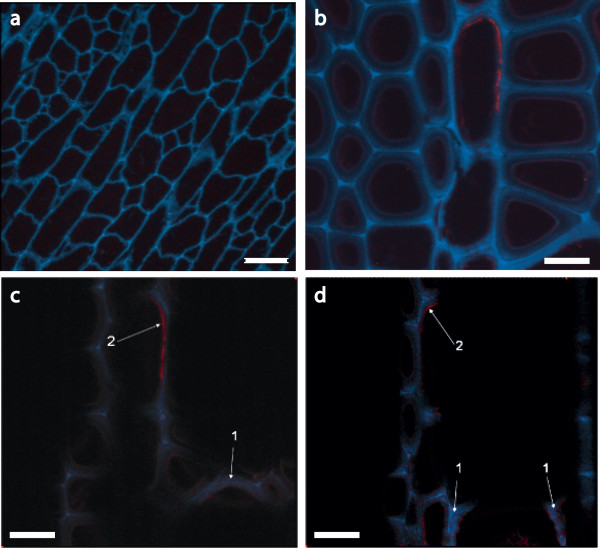
**Localization of E1 in transgenic tobacco shown in confocal microscopic images of sectioned WT and E1-containing tobacco**. All images were obtained by using immunofluorescence confocal laser microscopy. An E1cd primary antibody and an Alexa Fluor 488 antimouse secondary antibody were used and spectrally deconvoluted to show antibody in red and nonspecific autofluoresence in blue. **(a) **Negative control WT tobacco confocal image stack at ×600 magnification. Note the absence of E1 antibody staining in this image (red). **(b) **E1cd-transformed tobacco image stack at ×1,200 magnification. **(c and d) **Individual sections of the confocal image stack presented in **(b) **illustrate that E1cd antibody stained E1cd-transformed tobacco. Arrows indicate **(1) **deconvoluted E1 antibody signal present within a tobacco cell wall section (red) and **(2) **E1 antibody staining present on the inside of a cell wall (red). Blue coloring denotes the residual plant autofluorescence signal. Scale bar in **(a)**, 25 μm; original magnification ×600. Scale bars in **(b-d)**, 50 μm; original magnification, ×1,200.

Imaging cross-sections of both tobacco and maize plant cell walls obtained by anti-E1 immunomicroscopy clearly showed differences in the localization of E1 between these two plant types. The transgenic E1 is broadly distributed throughout the sclerenchyma-type cell walls of maize (Figure 8), whereas the E1 tobacco shows specific localization to the inner surface of the cell wall (Figure 9). In Figure 9, we can see localization of the E1 signal to interior regions of the plant cell wall. This result was expected, as E1 was expressed under the control of the CaMV 35S constitutive promoter and was targeted to the apoplast using the soybean variant-specific protein VSPβ secretion signal peptide.

## Discussion

We report here that tobacco and maize expressing heterologous E1 endoglucanase from *Acidothermus cellulolyticus *were observed to become less recalcitrant compared to wild-type biomass when subjected to pretreatment and post-pretreatment enzymatic hydrolysis. This reduction in recalcitrance was manifest through lower severity requirements to achieve comparable levels of conversion to wild-type biomass. Our studies indicate that the decreased recalcitrance was not due to post-pretreatment residual E1cd activity and could not be reproduced by the addition of exogenous E1cd to the biomass prior to pretreatment, indicating that the expression of E1cd during cell wall construction altered the inherent recalcitrance of the cell wall. In our experiments, expression of E1cd in maize increased the digestibility of biomass cell walls after pretreatment at expression levels far below those normally required for efficient E1 hydrolysis of cellulose. It also enabled lower-severity pretreated corn stover to become as digestible as higher-severity pretreated stover with the same enzyme loadings. In addition to decreased pretreatment severity requirements, the expression of E1 in corn stover is likely to enable decreased enzyme loadings for hydrolysis, decreased inhibitor formation and decreased degradation product formation. This effect is also likely to extend to other endoglucanases, especially those from glycoside hydrolase family 5.

We observed a shared phenomenon when expressing an endoglucanase in both corn stover and tobacco; that is, E1-expressing plants are more easily saccharified by cellulase enzymes following various severity pretreatments. Our results indicate that E1 expression in both corn and tobacco enabled reduction of the required pretreatment severity to permit the same level of sugar conversion as was obtained with higher-temperature pretreatments of wild-type samples. The possibility of reducing pretreatment severity is highly relevant to the biofuels industry because of the potential cost savings from lower energy and enzyme usage, reduced inhibitor formation and sugar losses, decreased catalyst addition and lower costs of facility construction.

Throughout the digestion experiments, transgenic E1 biomass, whether stover or tobacco, was shown to be either more digestible than the wild type after identical pretreatment or as digestible as more severely pretreated wild-type biomass. For example, with the 100 mg of enzyme per gram of biomass digestions, the yield of glucose from E1-expressing transgenic corn stover pretreated at 140°C appeared to be comparable to the yield from nontransgenic corn stover pretreated at about 170°C (Figure 5). This effect was even more distinct at lower enzyme loadings (15 mg of protein per gram of biomass; Figure 4). Tobacco also showed that transgenic expression of E1 benefited enzymatic conversion, although this effect was more pronounced at lower severities (Figure 3). As incubation of the biomass after the addition of exogenous E1 before pretreatment did not have any discernible impact on enzymatic conversion, we conclude that the effect is not due to residual post-pretreatment E1 activity. Also, as the incubation time was fairly long, the exogenous E1 would be expected to cleave some of the cellulose before pretreatment, although presumably the accessibility would be limited to outer layers of cellulose.

Our work in extracting E1 from the cell walls and quantifying it by Western blot analysis indicated that the E1 protein is tightly associated with the cell wall. Our normal extraction protocol of 100% ethylene glycol (as used in the cel7a purification method) did not remove detectable amounts of E1 from the biomass even after increasing the extraction temperature to ~96°C. Treatment by boiling with NuPAGE LDS sample buffer did remove E1 from the cell walls, indicating that the transgenic E1, even though it lacks a carbohydrate binding module, was tightly associated with the cell wall.

Imaging of plant cell wall sections by anti-E1 antibody localization clearly showed the presence of E1 throughout the cell wall, specifically in the thicker sclerenchyma cells of the rind and vascular bundles. Stover samples showed much higher levels of intrawall E1 deposition than did the tobacco samples, which seemed to have more E1 localized to the inner layers of the cell wall (Figures 8 and 9). This may indicate that measured E1 levels were higher in tobacco simply because the E1 was easier to extract. It may also explain why the tobacco conversion enhancement due to E1 expression was not apparent at the highest pretreatment severity; that is, the innermost layers of the tobacco cell wall were not as disrupted by pretreatment as were those of stover, which had more broad distribution of E1 throughout the cell wall. It is unclear from this work whether the localization was due to a preference of E1 to bind to a particular wall type, to a difference in cell-type expression or to some other variable (such as cell wall-type permeability to antibody). Additional maize transformation vectors have been generated in which E1 is driven by other strong promoters and fused to alternative signal peptides to target accumulation in the vacuole and endoplasmic reticulum, and these constructs will be used to generate additional transgenic lines for study. Entrained E1 may enhance the conversion, but only to the extent that it is distributed throughout the cell wall. Broader distribution of E1 in the stover cell wall interior could have allowed more rapid and complete digestion, which is indicated by the increased extent of conversion of E1 stover compared to E1 tobacco under identical high-enzyme loadings. Without the increased pretreatment efficacy from intercalated E1, the tobacco digestions all may have simply reached their limit at the 170°C pretreatment condition. Localization of cellulases using immunocytochemistry followed by transmission electron microscopy as described by Donohoe *et al. *[17] supports this idea, as cell wall disruption and enzyme penetration are directly proportional to pretreatment severity.

Our pretreatment, digestion, and imaging results lead us to believe that the cellulose in the E1 stover and tobacco has been modified by the expression of E1 during plant growth, biomass storage or both. On this basis, we conclude that the increased enzymatic conversion of the transgenic E1 biomass is due to the intrawall (and possibly intracellulose microfibril) localization of the E1 protein resulting from expression during cell wall formation. This conclusion is supported by the observation that wild-type corn stover treated with exogenous E1 prior to pretreatment was not as digestible as the transformed stover. We believe that this result was due to the ability of transgenic E1 to access more cell wall compartments containing cellulose and perhaps even the cell membrane-cell wall space occupied by the nascent (growing) cellulose microfibril during cellulose synthesis than was possible with the simple external addition of enzyme. This idea is supported by our imaging data showing that E1 expressed during plant growth is targeted to the cell wall, where it may work by nicking the cellulose chains as they are formed or laid down, resulting in either less crystalline cellulose material or simply more free chain ends for cellobiohydrolases, which could result in increased conversion. It is not known whether the reduced activity of this thermal tolerant enzyme at growth condition also benefited this effect. Alternatively, expression of the E1 enzyme in plant tissue may likely have led to increased conversion through a nonenzymatic effect, such as the ability to bind strongly to polysaccharides. By simply binding to the cellulose microfibril during plant growth and development, E1 may decrease the recalcitrance of the cell wall to chemical pretreatment. The comparable results between tobacco and corn expressing E1 suggest that low-level expression of E1 or other endoglucanases in plants may be a general phenomenon by which the conversion of biomass may be improved.

## Materials and methods

### E1 gene and protein

The *E1 *gene used to transform tobacco and maize was originally isolated from *A. cellulolyticus *and was later truncated to remove the carbohydrate-binding module and linker, resulting in a gene containing only the E1 catalytic domain (E1cd) [16]. E1cd was expressed and purified from *Streptomyces lividans *for use as a control as described previously [18].

### Tobacco

Tobacco was transformed according to standard methods using *Agrobacterium tumefaciens *[7], and the transformed plants grew well. We received the E55 tobacco (apo E1cd) seeds from Sandra Austin-Phillips at the University of Wisconsin-Madison [7], identified stable lines and grew the biomass under controlled greenhouse conditions.

To verify the presence of the *E1 *gene, the third or fourth leaf from the shoot apex was used for protein extraction. Leaf samples were harvested 2 to 3 hours into the light period, cut into approximately 1-cm^2 ^pieces and pooled for homogenization. An enzyme assay, sodium dodecyl sulfate (SDS)-polyacrylamide gel electrophoresis and Western blot analysis were carried out as described previously [19]. For estimation of E1cd in the samples, the ground biomass was extracted directly by boiling 20 mg of biomass in 100 μL of NuPAGE LDS sample buffer for 10 minutes and loading 20 μL of supernatant onto a NuPAGE 3-(N-morpholino)propanesulfonic acid gel (Invitrogen). After electroblotting onto polyvinylidene fluoride (PVDF), the blot was analyzed for E1cd using mouse anti-E1 monoclonal primary antibody and goat antimouse secondary antibody. E1cd purified from expression in *Streptomyces lividans *was used as a quantitative control for comparison with a dilution series of the E1 tobacco extracts (Figure 1).

### Maize

Maize was used as a model lignocellulosic substrate, as corn stover (the nongrain parts of the harvested plant) is a strong potential biofuels feedstock. Ransom *et al. *[11] produced E1cd-transformed maize in which E1 was driven by the 35S CaMV promoter and targeted to the apoplast. E1 corn was grown to maturity under greenhouse conditions, then allowed to dry down in the greenhouse before being harvested. Individual plants were screened for E1 activity, and initial studies focused on two transgenic lines designated E1-1 and E1-7, which showed high and low E1 activity, respectively. Stalks were further air-dried to a moisture content of approximately 10%, and the entire stalk (that is, stover) was milled to pass a 20-mesh screen.

E1 expression was estimated by Dot blot assay (not shown) as well as Western blot analysis (Figure 1). For the Dot blot assay, 10 mg of 80-mesh stover was extracted three times in 100 μL of 2% SDS by boiling it for 10 minutes, centrifuging, reextracting the solids and combining all supernatants. For the Western blot analysis, ~5 mg of biomass was directly extracted with 50 μL of NuPAGE LDS sample buffer by boiling it for 10 minutes. After running electrophoresis, the gel was electroblotted onto a PVDF membrane using the Invitrogen NuPAGE blotting apparatus. Both Dot blot and Western blot analyses were visualized using the antimouse Western Breeze Chromogenic Kit (Invitrogen) with anti-E1 mouse monoclonal antibody as the primary antibody.

The activity of E1 in milled stover and tobacco was estimated on the basis of MUC activity. In a 16 × 100-mm test tube, we added 10 mg of 80-mesh biomass, 200 μL of 1.0 M sodium acetate, pH 5.0, and 1,600 μL of H_2_O. After heating the sample to 84°C for 5 minutes, 200 μL of 2.0 mg/mL MUC substrate was added to the reaction mix. Each sample was analyzed in triplicate. Samples of 100 μL were taken at 0.0, 0.5, 1.0 and 2.0 hours (for stover) or every minute for 15 minutes (for tobacco), quenched with 200 μL of 1.0 M Na_2_CO_3 _and fluorescence was measured at 360-nm excitation/465-nm emission in a Tecan GENios microplate reader (MTX Lab Systems, Vienna, VA, USA). E1cd levels in the stover were estimated by comparison of activity to purified *S. lividans*-expressed E1cd activity under the same conditions (Figure 2).

### Pretreatment

Both tobacco and stover rind were milled to 20 mesh in a Wiley knife mill(Thomas Scientific, Swedesboro, NJ, USA) and analyzed for sugar composition according to National Renewable Energy Laboratory (NREL) standard laboratory protocol NREL/TP-510-4618 [20]. Samples were vacuum-impregnated with water at 10% (wt/vol) biomass. Exogenous E1 was added to select biomass samples at 0.39 mg of protein per gram biomass, and then all samples were incubated overnight at 80°C. Pretreatment was performed in gold-plated batch reactors (15 mL) with 1% (wt/vol) sulfuric acid at 5% (wt/vol) solids loading. The reactor was rapidly heated to 110°C, 140°C or 170°C within approximately 5 minutes by immersion in a sand bath at 10°C to 20°C above the target temperature. The reactor was then rapidly transferred to a second sand bath maintained at the target temperature, where it remained for 10 minutes. The reactor was removed from the sand bath and rapidly cooled by immersion in ice water. In the case of tobacco, a secondary control consisting of 50% wild-type and 50% E1 tobacco was mixed and pretreated as above. Following pretreatment, all solids were washed with distilled water until the pH of the rinse was measured to be above 4.0.

Because of sample size limitations of the pretreatment reactor chambers, all samples were pretreated in triplicate at each temperature, then the replicates were pooled and washed with water until the pH was greater than ~4.5. Pretreatment replicates were from the same bulk batch of stover or tobacco. Three aliquots of each of the pooled, washed solids were taken for analysis by enzyme digestion. Duplicate samples were used for the compositional analysis of each sample.

### Analysis of pretreated solids

To provide a basis for the maximum theoretical sugar yield achievable from each substrate during enzymatic hydrolysis, portions of the washed, pretreated solids were dried and subjected to the standard two-stage sulfuric acid hydrolysis method for determining structural carbohydrates in lignocelluloses as described by Sluiter *et al. *[20]. The glucan and xylan content of each pretreated sample was calculated from the glucose and xylose released as described by Sluiter *et al*.

### Enzymatic saccharification

Saccharification of all pretreated and washed biomass slurries was conducted at pH 5.0 in 50 mM citrate buffer with 0.01% sodium azide added as an antimicrobial agent. One percent (wt/vol) slurries of pretreated solids were hydrolyzed with either a low (15 mg of cellulose per gram of biomass) or high (100 mg of cellulose per gram of biomass) loading of a commercial cellulase (Spezyme CP) for 72 hours at 50°C. Additional saccharifications of transgenic maize were conducted with a purified cellobiohydrolase, cel7A from *Trichoderma reesei*, to assess the effect of plant-expressed E1 on the ability of this exocellulase to digest cellulose. The cel7A was purified from a commercial *T. reesei *cellulase (Spezyme CP) by using column chromatography (dual anion exchange followed by affinity on cellulose and size-exclusion chromatography[21]) and loaded at 15 mg of protein per gram of biomass. All reactions were supplemented with 5.0 mg of β-glucosidase per gram of biomass to limit cellobiose inhibition. Whole cellulase digestions were supplemented with Novo188 (Novozymes NA Inc., Franklinton, NC, USA), while Cel7a digestions were augmented with purified *Aspergillus niger *β-glucosidase. Glucose concentrations in the hydrolysates were determined on a Jasco high-performance liquid chromatography system (Jasco, Great Dunmow, UK) running a lead-based Shodex sugar column (model SP0810; Kawasaki, Japan) heated to 80°C with water flowing at 0.6 mL/min as the mobile phase. All saccharification experiments shown in Figures 3-7 were run in triplicate and error bars show standard deviations.

### Imaging methods

Several 3-mm-wide sections of unpretreated tobacco rind were cut and dehydrated in a graded ethanol series. LR White resin (Electron Microscopy Sciences, Hatfield, PA, USA) was serially added, and the sections were left overnight in 100% LR White. For optimal infiltration, samples were placed in a vacuum microwave oven for 1 and 5 minutes during each ethanol and resin concentration rinse, respectively. After 2 days, the samples were transferred to flat-bottomed "BEEM" capsules (Electron Microscopy Sciences, Hatfield, PA, USA) and filled with 100% LR White. Polymerization occurred overnight in a vacuum oven set to 60°C. Resin blocks were trimmed with a Leica EM Trim trimmer (Leica Microsystems, Bannockburn, IL, USA), and 2.0-μm sections were cut using glass knives and a Leica Ultracut UCT ultramicrotome (Leica Microsystems). Glass knives were made from a 6-mm glass bar and cut on a LKB Bromma 7800 KnifeMaker (Bromma, Sweden).

Confocal laser scanning microscopy images of E1 antibody-stained samples at ×200, ×600 or ×1,200 magnification were obtained using a Nikon Eclipse 90i Spectral Laser Confocal Microscope (Nikon Instruments, Inc., Melville, NY, USA). For confocal microscopy, 2.0-μm thin sections of maize rind were blocked with 1% (wt/vol) bovine serum albumin for 45 minutes to minimize nonspecific binding, washed five times with phosphate-buffered saline (PBS) and incubated with mouse anti-E1cd monoclonal antibody for 45 minutes. After washing with PBS, an Alexa Fluor 488-conjugated antimouse immunoglobulin G secondary antibody (Invitrogen) was added to localize bound anti-E1cd. Excess secondary antibody was washed using PBS. Autofluorescence and nonspecific signal were subtracted out using Nikon EZ-C1 software (Nikon Instruments, Inc. Melville, NY, USA) to deconvolute the Alexa Fluor signal from the background plant autofluorescence.

## Competing interests

Some of the work presented in this paper was also filed in U.S. Provisional Patent 61/074,497. MEH is an editor of this journal.

## Authors' contributions

RB carried out the imaging studies and the pretreatment of the samples, participated in the digestion studies and evaluation of E1 and drafted the manuscript. MS carried out the digestion studies. TV participated in the pretreatment of samples. MH participated in the design of the study. DL provided the plant material. MB and SD conceived of the study and participated in its design and coordination. All authors read and approved the final manuscript.
